# Application of novel burst wave lithotripsy and ultrasonic propulsion technology for the treatment of ureteral calculi in a bottlenose dolphin (*Tursiops truncatus*) and renal calculi in a harbor seal (*Phoca vitulina*)

**DOI:** 10.1007/s00240-023-01515-6

**Published:** 2024-01-08

**Authors:** Arturo E. Holmes, Ben H. Chew, Robert Laughlin, Jean Buckley, Erica Kiewice, Michael J. Dancel, David Blasko, Victor K. F. Wong, Abdulghafour Halawani, Kyo Chul Koo, Doug Corl, Paul Fasolo, Oren Levy, Jeff Thiel, Michael R. Bailey, Jammy Eichman, Jennifer M. Meegan, Martin Haulena

**Affiliations:** 1 Dept. of Urology, University of Washington School of Medicine, Seattle, WA USA; 2 Department of Urological Sciences, University of British Columbia, Stone Centre at Vancouver General Hospital, British Columbia, Vancouver, Canada; 3 The Mirage Hotel, Hard Rock International, Las Vegas, NV USA; 4 Department of Urology, King Abdulaziz University, Jeddah, Saudi Arabia; 5 Department of Urology, Yonsei University College of Medicine, Seoul, Republic of Korea; 6 SonoMotion, Inc, San Mateo, CA USA; 7 Ctr. for Industrial and Medical Ultrasound, Applied Physics Lab, Univ. of Washington, Washington, USA; 8 National Marine Mammal Foundation, San Diego, CA USA; 9 Vancouver Aquarium, Vancouver, BC Canada

**Keywords:** Lithotripsy, Urolithiasis, Ultrasound

## Abstract

Marine mammals may develop kidney stones, which can be challenging to treat. We describe burst wave lithotripsy (BWL) and ultrasonic propulsion to treat ureteral calculi in a 48-year-old female bottlenose dolphin (*Tursiops truncatus*) and to reduce renal stone burden in a 23-year-old male harbor seal (*Phoca vitulina*). BWL and ultrasonic propulsion were delivered transcutaneously in sinusoidal ultrasound bursts to fragment and reposition stones. Targeting and monitoring were performed with real-time imaging integrated within the BWL system. Four dolphin stones were obtained and fragmented ex vivo. The dolphin case received a 10-min and a 20-min BWL treatment conducted approximately 24 h apart to treat two 8–10 mm partially obstructing right mid-ureteral stones, using oral sedation alone. For the harbor seal, while under general anesthesia, retrograde ureteroscopy attempts were unsuccessful because of ureteral tortuosity, and a 30-min BWL treatment was targeted on one 10-mm right kidney stone cluster. All 4 stones fragmented completely to < 2-mm fragments in < 20 min ex vivo. In the dolphin case, the ureteral stones appeared to fragment, spread apart, and move with ultrasonic propulsion. On post-treatment day 1, the ureteral calculi fragments shifted caudally reaching the ureteral orifice on day 9. On day 10, the calculi fragments passed, and the hydroureter resolved. In the harbor seal, the stone cluster was observed to fragment and was not visible on the post-operative computed tomography scan. The seal had gross hematuria and a day of behavior indicating stone passage but overall, an uneventful recovery. BWL and ultrasonic propulsion successfully relieved ureteral stone obstruction in a geriatric dolphin and reduced renal stone burden in a geriatric harbor seal.

**Supplementary Information:**

The online version contains supplementary material available at 10.1007/s00240-023-01515-6.

## Introduction

Bottlenose dolphins (*Tursiops truncatus*) under professional care are susceptible to developing kidney stones composed of 100% ammonium urate (NH_4_U) [1, 2]. Kidney stone disease in dolphins has been reported to cause azotemia, hematuria, reduced renal function, renal obstruction, and renal failure [3–5]. Case reports on seals describe kidney stone formation, compromised kidney function, and death resulting from stones and stone treatment. [6, 7]

Urologists are infrequently called away from their human patients and asked to remove or prevent life-threatening stones causing urinary tract obstructions in these and other animals [3]. Traditional methods of calculi treatment including ureteroscopic laser lithotripsy are often ineffective in marine mammal species because of anatomic adaptations including multilobulated reniculate kidneys with numerous collection systems. Recent reports have described successful and unsuccessful outcomes and challenges associated with surgical treatment options for marine mammals, warranting continued exploration for potential minimally invasive options [3]. Burst wave lithotripsy (BWL) and ultrasonic propulsion have recently been developed based on shock wave lithotripsy research [8] to noninvasively break stones and reposition fragments to facilitate clearance in humans without requiring general anesthesia [9–18]. Most relevantly, 89% of human distal ureteral stones treated with BWL and/or ultrasonic propulsion passed in 3.9 days [17].

Here, we report the use of these novel, noninvasive human technologies, BWL and ultrasonic propulsion, to clear partially obstructing ureteral stones in a female bottlenose dolphin and to reduce a renal stone burden in a male harbor seal.

## Methods

### Ex vivo* stone fragmentation*

To confirm that BWL would be able to fragment ammonium urate stones, renal stones from previous bottlenose dolphin cases were obtained with permission from the U.S. Navy Marine Mammal Program. The stones were soaked for > 48 h in degassed water to rehydrate. BWL was attempted using one 10-mm stone with the University of Washington (UW) system at 5 MPa peak negative pressure; and 3 stones (6, 8, and 15 mm) with the SonoMotion BreakWave system (SonoMotion, San Mateo, CA) at 8 MPa peak negative pressure. The UW system had lower pressure because skin to stone distance on ultrasound was 7.5 cm which was deeper than the focus on the UW transducer; the SonoMotion system uses 3 different probes to cover the full range of skin to stone depths encountered in humans with the dolphin and harbor seal having a very similar range. The maximum acoustic exposures for BWL and ultrasonic propulsion measured are listed in Table 1 [9, 12, 18]. Fragmentation was filmed with a camera with the SonoMotion system and was observed with ultrasound in the UW system. The UW stone was removed every 5 min, sieved through a 2-mm mesh, and weighed, and all fragments were returned to the tissue calyx phantom. The technique and phantom were the same as described in Ref. 9 with the transducer facing down in the water bath. The stones were held in a 60-mm diameter by 70-mm tall cylindrical tissue phantom made of polyvinyl chloride (PVC) (Soft Baits, Do-it Molds, Denver, IA). The phantom contained a depression or cavity in the top surface. The depression dimensions were 15-mm diameter by 40-mm depth. A 3-mm thick disk of PVC was used to cover the cup.

**Table 1:** Maximum free-field acoustic exposures for ultrasonic propulsion and BWL

Ultrasound therapy	Peak negative pressure at stone	Burst duration	Burst repetition rate	Ensemble (of bursts) duration	Total exposure (All ensembles)
Ultrasonic propulsion	2.4 MPa	25 ms	20 Hz	3 s	30 min
BWL	5 MPa (dolphin) 8 MPa (seal)	0.2 ms	10 Hz	30 s	

### Dolphin procedure

The dolphin case was a 48-year-old, female bottlenose dolphin with a chronic history of severe bilateral kidney stone burden and urinary and respiratory infection. At the time of BWL treatment, the dolphin was being treated for a chronic urinary tract infection, believed to be secondary to the extensive bilateral kidney stones and benign genital papilloma tumors at the level of the urethral opening. Supportive care and treatments included oral and intravenous fluids for hydration support, and intravenous antibiotics. The ureter stoen had been present and obstructing for a > 21 days at time of treatment. 

**Fig. 1:**
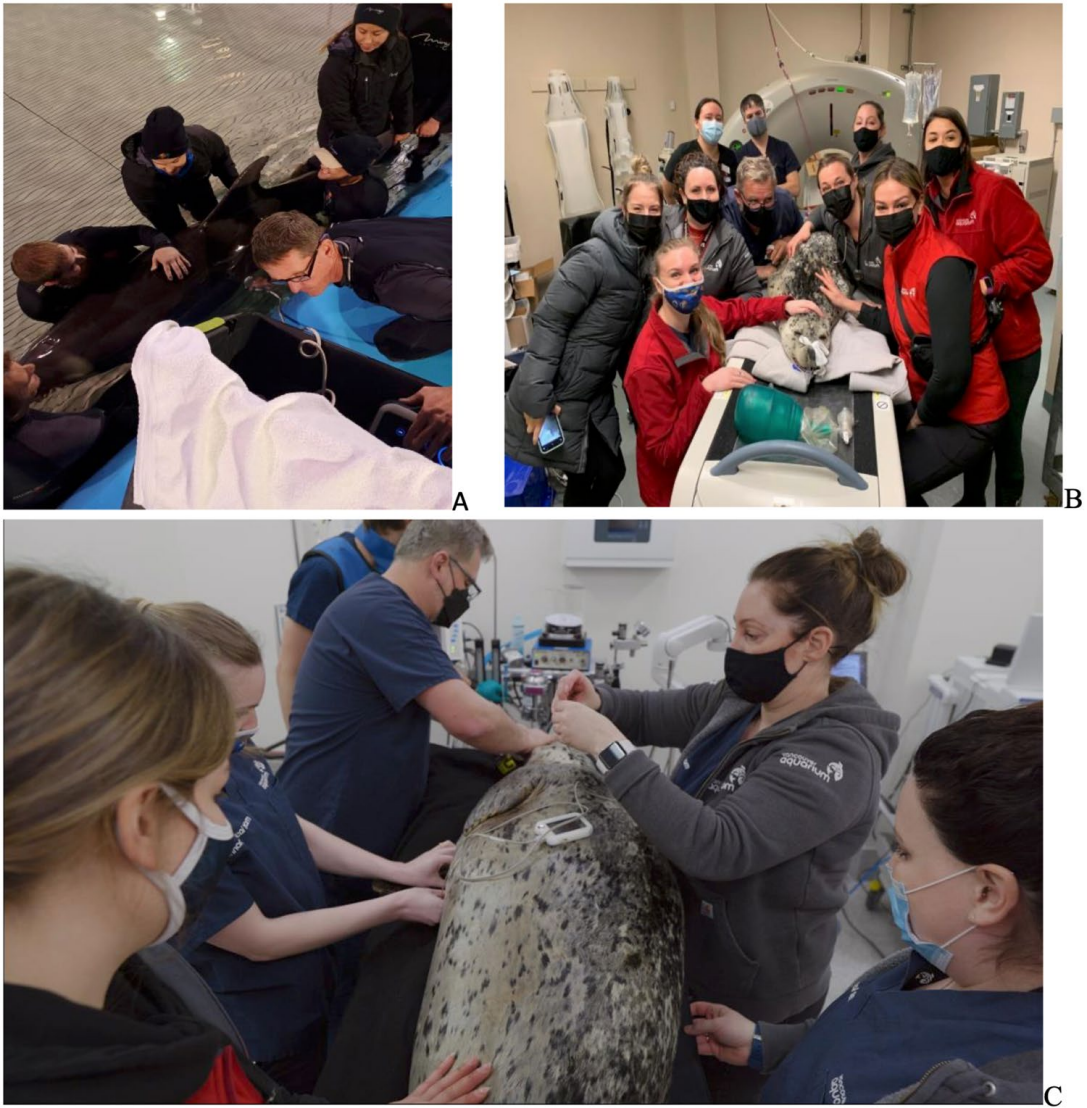
Photographs of the animal positing. **A** Ultrasound imaging of the dolphin in the isolation pool. It was possible to conduct the BWL therapy in this same arrangement, but the dolphin was moved out of the water and placed on a mattress poolside. The harbor seal is positioned for **B** CT examination and **C** the BWL procedure

Oral diazepam (15 mg) was administered approximately 1.5 h before the procedure to provide light sedation. The dolphin was then transferred into a stretcher and removed from the water and placed onto a thick padded foam mat for the procedure. Patient monitoring included continuous EKG, heart rate, and respiratory rates, and the patient was kept cool by continuously misting her skin with water. Patient positioning for the procedure was left lateral recumbency to optimize the acoustic window for locating the right ureter and ureteral stones and to reduce the distance between the outer skin layers and the stones. Ultrasound gel was applied to the skin, and the veterinarian or sonographer held the transducer on the dolphin’s skin, aligned the stone with the focus indicator using the real-time ultrasound image guidance which was recorded, and delivered individual bursts no longer than 3 s at a time for ultrasonic propulsion and 30 s for BWL. The UW BWL system was used [9, 14]. Follow-up ultrasound imaging was done with the dolphin in the pool as shown in Fig. 1.

### Harbor seal procedure

The harbor seal case was a 23-year-old male that presented with intermittent inappetence and clinical signs consistent with abdominal discomfort. Sonographic examination revealed multiple renal calculi in both kidneys. The extensive number and the location of nephroliths was confirmed via computed tomography (CT). Because of failure of treatment with allopurinol and a guarded prognosis based on a previous case (a similarly aged harbor seal in the same aquarium recently died of renal failure whereby a postmortem revealed extensive renal stone burden), alternative treatment methodologies were investigated. The seal was placed under general anesthetic and placed in the right lateral recumbent position to allow simultaneous access to the flank and penile urethra for simultaneous retrograde ureteroscopy and BWL (Fig. 1C, D). The BWL transducer was placed on the side just under the ribs after ultrasound gel was applied to the shaved fur of the seal [18]. Thirty minutes of BWL were delivered up. We focused on one < 20-mm sized region, as guidance for shock wave lithotripsy is to avoid larger stones in humans, [19] and the harbor seal’s ability to pass many fragments was unknown. Ureteroscopy was simultaneously attempted using a single use digital ureteroscope (LithoVue^™^, Boston Scientific, Marlborough, MA) through an 11/13Fr 46 cm ureteral access sheath (Navigator^™^, Boston Scientific, Marlborough, MA). A degradable ureteral stent (URIPRENE^™^, ADVA-Tec, South Carolina) was prepared for post-operative drainage that would not require subsequent removal.

## Results

### Ex vivo* stone fragmentation*

**Fig. 2:**
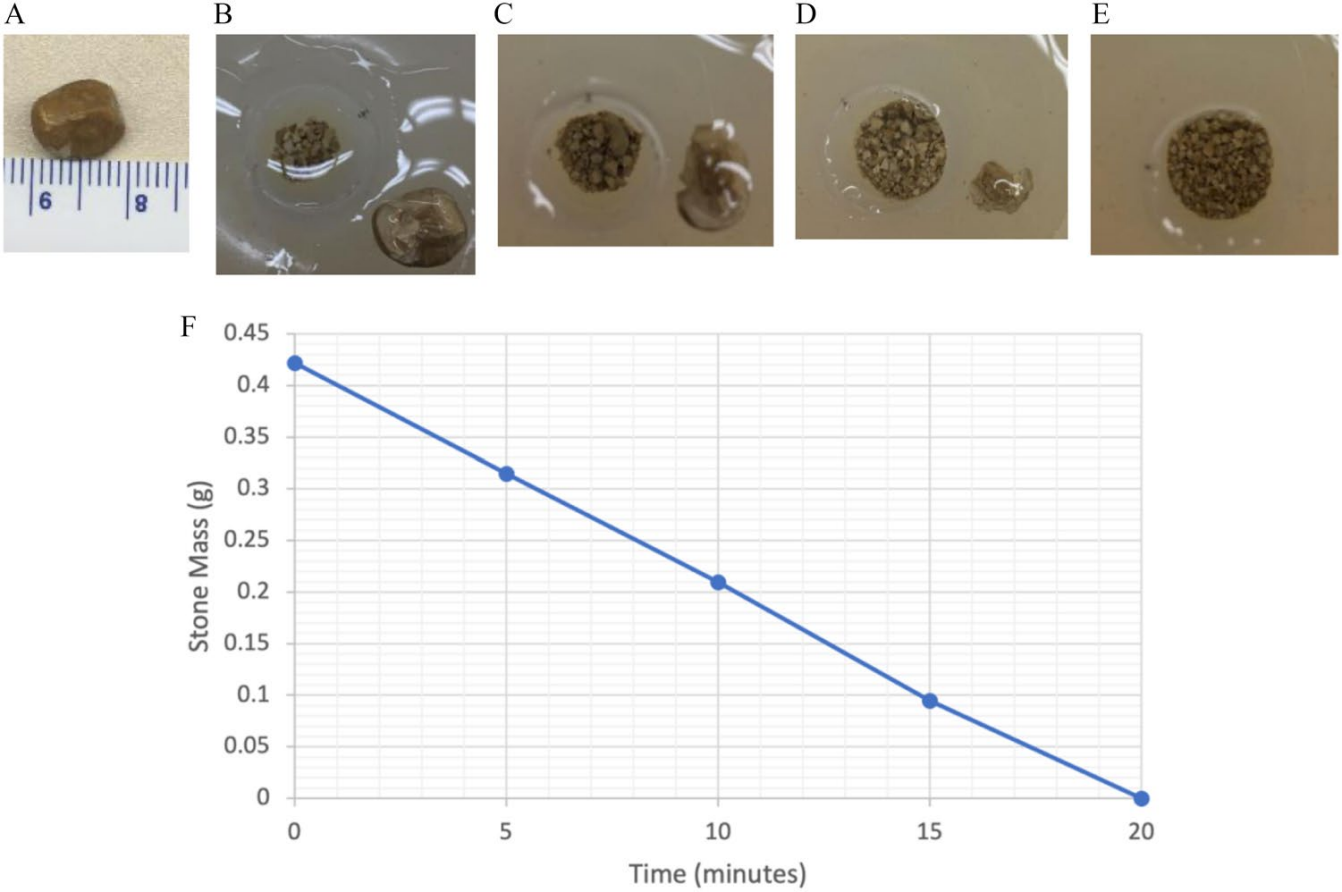
Fragmentation of a 10-mm dolphin stone that had been rehydrated in a small amount of water for one week. Photographs of remaining stone > 2 mm and the collection of fragments < 2mm in the treatment phantom at (**A**) time zero (pre-treatment), **B** after 5 minutes, **C** after 10 minutes, **D** after 15 minutes, and **E** after 20 minutes (the stone was completely fragmented). **F** A graph of the mass of any fragments > 2 mm over time. The dolphin stones broke by chipping off small passable fragments and not by cracking into larger pieces that might then obstruct

The 10-mm dolphin stone in the calyx phantom was completely fragmented within 20 min (Fig. 2) using 5-MPa BWL from the UW system. Supplemental video 1 shows the video of the screen during treatment resulting in a fragmented stone. Three other stones 6, 8, and 15 mm completely comminuted to less than 2-mm fragments in 1.5, 3.2, and 20 min with 8-MPa BWL from the SonoMotion system (Supplemental video 2).

### Dolphin procedure

**Fig. 3:**
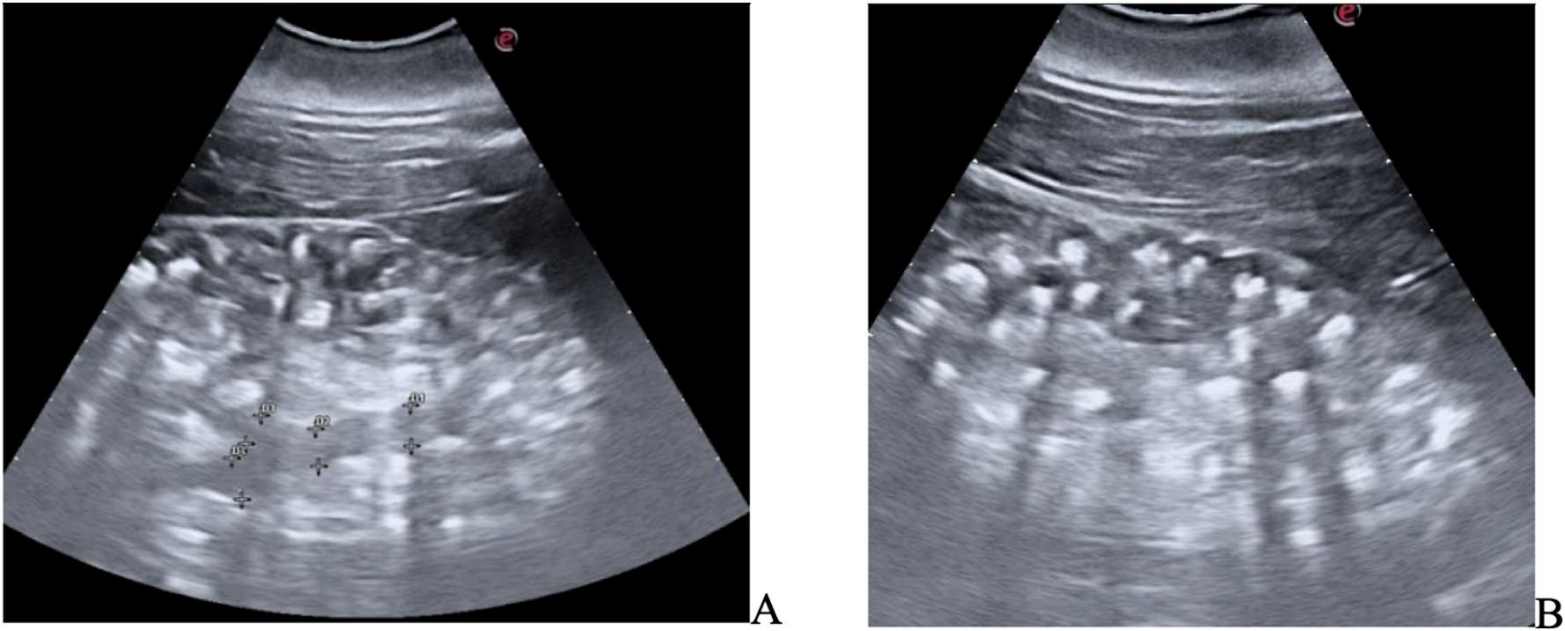
Resolution of dilation of the collecting duct in the right kidney (upper row) and renal stone burden. **A** on (post-procedure day 1), ultrasound images of the kidney show many stones and a dilated collecting duct. **B** By post-procedure day 15, the duct has relaxed as the obstruction has broken up and passed. Similar stone burden is seen in the harbor seal in fig. 6

There was no interference with bowel gas or ribs, and the stones were imaged from several angles within a large acoustic window. The stone position was very stable and did not move during breathing, except with infrequent large exhalations. Imaging showed a 10-mm stone and an 8 mm stone located in the right mid-ureter where the ureter crossed an artery similar to how human stones obstruct near where the ureter crosses the iliac vessels. Ultrasound showed distension of the right ureter proximal to the stones, as well as the collecting duct and the reniculated kidney. (Fig. 3).

**Fig. 4:**
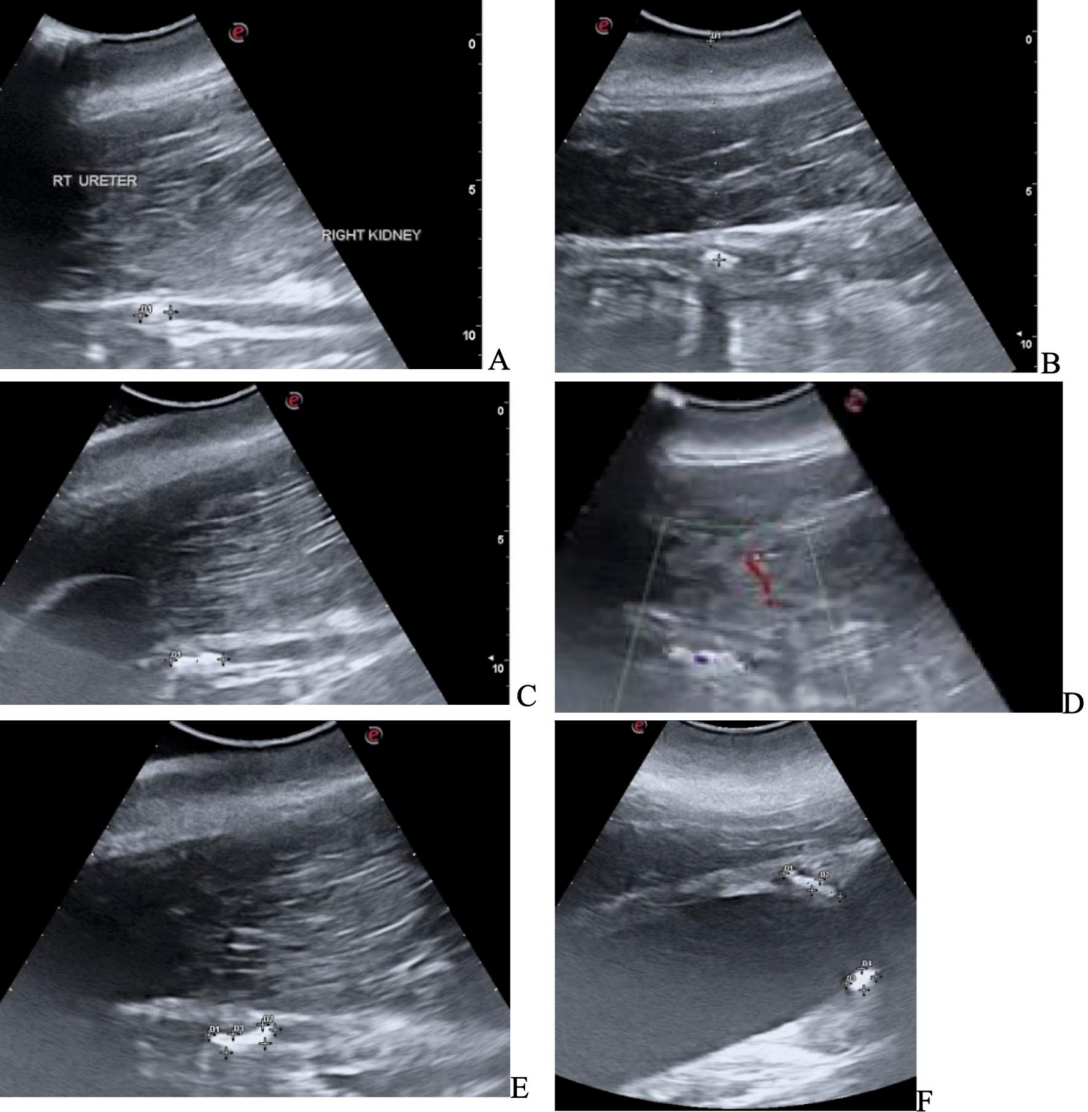
Timeline of dolphin ultrasound images. **A** 21 days prior to the procedure (Pre-procedure Day-21)—a single 10-mm right mid-ureteral stone is identified at a depth of 9–10cm. **B** 6days prior to the procedure (Pre-procedure Day-6), the stone still in the same location mid-ureter is able to be visualized with a 7.2cm skin to stone distance. **C** 1 Day prior to the procedure (Pre-procedure Day-1) a second 8mm stone was seen immediately proximal to the first stone. **D** Immediately post-procedure (Day 0), the stone appears as a line of broken fragments after treatment. **E** 1 Day post-procedure (Day +1), the fragments have shifted caudally getting past where the ureter crosses an artery. **F** 9 Days post-procedure (Day+9), the fragments migrated to the right ureteral orifice. 10 Days post-procedure (Day+10), the right ureteral fragments were no longer visible

Two BWL procedures were performed for 10 min and 20 min within 24 h. Frequently the operator (veterinarian or sonographer) switched from the treatment/imaging probe to a verterinary imaging probe to provide visual updates and confirm targeting. On the very first BWL pulse, the dolphin reacted. However, vitals remained normal, and animal remained alert and stable. Following that, the patient tolerated the treatments well without movement. For short periods during the treatment, her heart rate elevated from 70 to 90 beats per minute, but she remained stationary to allow treatment. The stones appeared to fragment, and ultrasonic propulsion pulses applied to the collection appeared to reposition and spread the fragments. Figure 3 shows the before and after images with the veterinary ultrasound machine. Rather than two large ureteral stones seen prior to the treatment, post-treatment there appeared to be a wider region of smaller fragments within the ureter (Fig. 4).

The afternoon and evening of treatment day, the dolphin was seen to be “hunching” or straining in what presumably was an attempt to pass the ureteral stones. The day after treatment, there appeared to be ultrasonographic evidence of slight thickening of the ureteral wall (Fig. 4E) and the stones migrated caudally a few millimeters. Fragments clustered together were now visible distal to the iliac artery. Based on the behavioral signs consistent with passing stones, the ultrasonographic evidence of potential mild aggravation to the ureteral wall, and indication of stone fragmentation and movement, further observation and conservative management were elected. From this point forward, the dolphin improved her appetite, was less lethargic, although there was not a full return of energy and her blood work and azotemia did not recover to normal levels. Her blood urea nitrogen (BUN)/creatinine levels were 63/5.3 on Day 0, 75/4.9 on Day + 2 and 77/4.1 mg/dL on Day + 15.

**Fig. 5:**
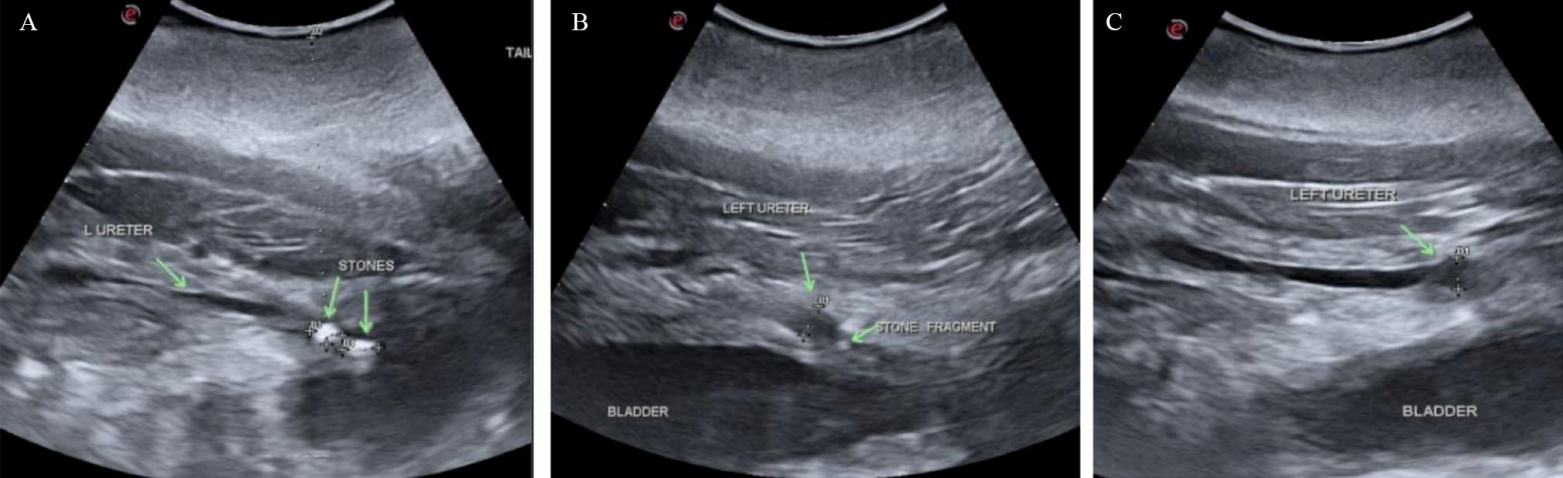
Identification of two left ureteral stones on post-procedure (Day+10), at the ureteral orifice complicated recovery. **A** Post-procedure (Day+10), two stones are seen at the left UO. They can also be seen in Fig. 4f. **B** post-procedure (Day+10), only punctate fragment remained at the left UO. **C** However, the ureter remained dilated and had not yet relaxed

On post-procedure day 9, ultrasound showed the fragments migrated distally and were seen at the ureteral orifice (UO). On day 10, the right ureter fragments had passed, but two 6-mm stones were discovered at the left UO, which passed the next day. Figures 3 and 4 show the before and after dilation as well as the large stone burden in the right, and Fig. 5 shows the stones and ureteral dilation on the left. On day 13, the bilateral hydroureter had resolved, azotemia, appetite and attitude also improved. Unfortunately, despite successful BWL treatment and aggressive supportive care, the animal succumbed to complications associated with her chronic illnesses and infections, and ultimately died from causes determined to be unrelated to the BWL procedure.

### Harbor seal procedure

Retrograde ureteral access took a prolonged time because of the tortuous urethra and ureter. After 90 min, the ureteroscope reached the renal pelvis through a ureteral access sheath, but the harbor seal became unstable under anesthesia (labile blood pressure), and all procedures were finished at that time.

For 30-min during the ureteroscopy attempt, a radiologist, urologist, and veterinarian each took turns operating the BWL. Image quality was very good without further tuning of this system from human use (Supplemental video 3). One 10-mm stone in the lower region of the kidney was the primary target. Additional stones in the vicinity also entered the target region with the seal’s respiration. Real-time ultrasound image guidance from the SonoMotion system showed complete fragmentation of the primary stone. Numerous stones in the vicinity of the main stone targeted also appeared to be reduced substantially.

**Fig. 6:**
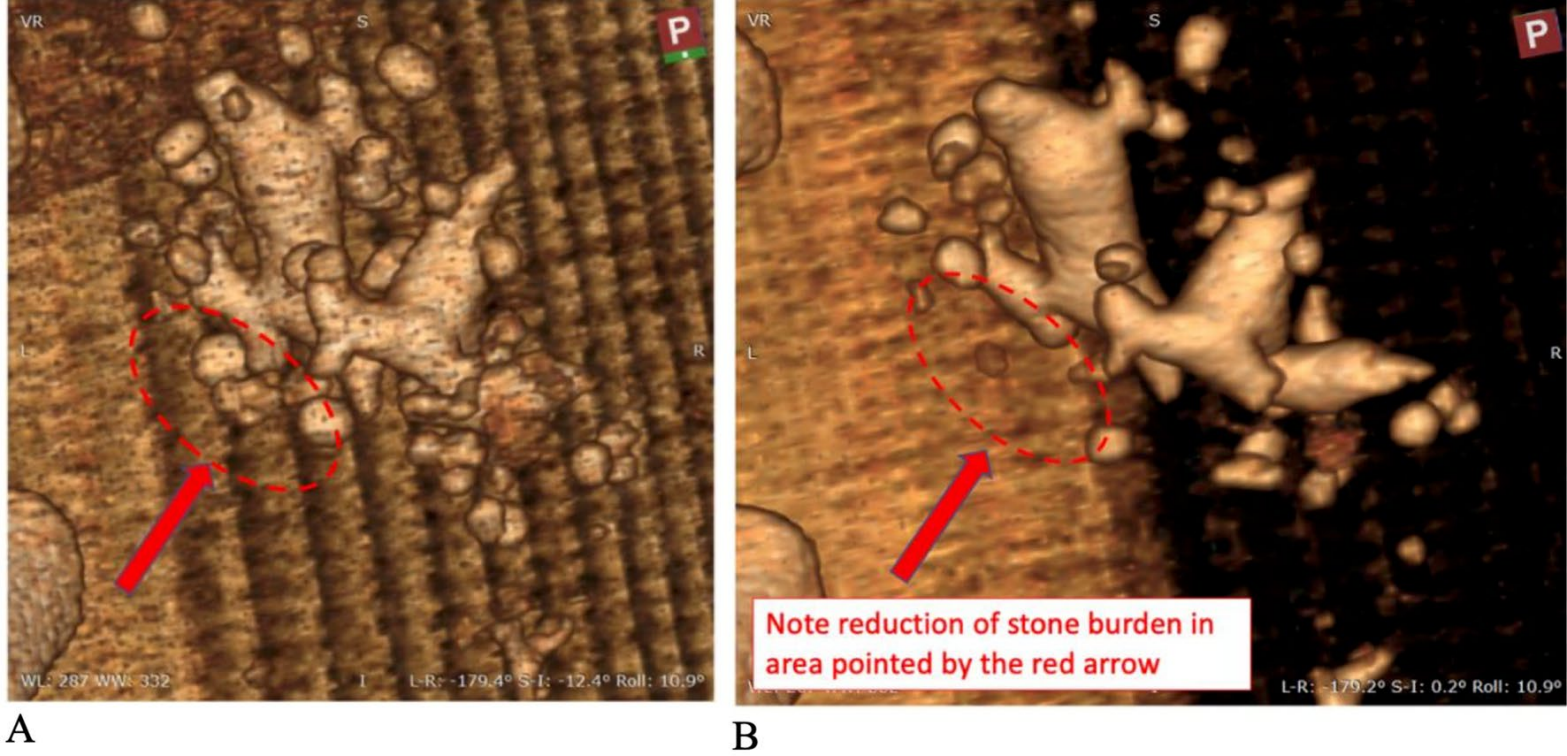
Before and after three-dimensional reconstruction of the CT image of the treated region in the harbor seal. The red arrow point to the treated region. The oval shows the treated area nearly 2 cm in length and the stones that were broken and passed

The harbor seal had some hematuria and mild abdominal pain post-operatively. All clinical signs resolved within 4 days after the procedure. The primary stone and adjacent stones in 4-cm region were absent in the post-operative CT exam 21 days after the procedure (Fig. 6).

## Discussion and conclusions

BWL and ultrasonic propulsion successfully relieved ureteral stone obstruction in a geriatric dolphin and reduced renal stone burden in a geriatric harbor seal.

Dolphins and harbor seals have the potential to form ammonium urate stones, a form of uric acid stones [1–7]. These grow to fill their reniculated kidneys [2]. Dolphin and harbor seal kidneys lack a true renal pelvis and dilation of the ureter and collecting duct from an obstructing stones, as well as the stones themselves, can be detected on ultrasound [2]. Obstruction of part of the kidney by renal stones and the entire kidney by ureteral stones is possible and can lead to azotemia [3]. It has been our clinical experience that marine mammals may be expected to pass ureteral stones up to 10 mm in size, and American Urological Association (AUA) guidelines expect passage for stones up to 5 mm in humans [19]. Ureteroscopy to remove obstructing ureteral stones has been successfully performed in several female dolphins; however, successful treatment in a male dolphin has not yet been achieved, and renal stones in harbor seals may be impossible to remove via ureteroscopy because of the tortuous anatomy. [3]

Here, minimally invasive BWL and ultrasonic propulsion were used to treat obstructing ureteral stones and to reduce renal stone burden in two marine mammals with mild adverse events. Uric acid stones in marine mammals and in humans break readily within minutes with BWL even at very low-pressure output levels [11]. BWL has been shown to fragment a target stone by producing smaller than 2-mm fragments until complete comminution occurs, and this may reduce the risk of multiple large fragments causing *Steinstrasse* and enable prophylactic reduction of sizable stone burdens seen in even young otherwise healthy marine mammals [11]. In addition, repeated BWL procedures are possible to remove the extensive stone burdens impacting the health of geriatric marine mammals. In humans, following SWL guidelines, BWL and propulsion retreatments have been delayed at least a month, but here the dolphin received two treatments within 24 h and successful clearance of the stone.

A urologist in both marine mammal cases was needed to diagnose the need to intervene. With the ureteral stone, the urologist’s judgement assessed whether the stone was impeded in the ureter because of the crossing vessel or for some other reason. And with renal stones, the urologist’s clinical judgment directed prophylactic intervention after another harbor seal passed from kidney failure with an extensive stone burden and was the best guide of how much stone should be treated and could be passed without obstruction. Once intervention was indicated, BWL was performed by several operators with different professional training and various levels of BWL training. All observers could successfully target the stone and assess effectiveness. A veterinarian with skill in ultrasound was helpful to give confidence in the stone identification and targeting, but ultimately, ultrasound images of kidney and ureteral stones were straightforward to interpret. Similarly, stone breaking was identifiable in the image during BWL as fragments moved and separated from the main stone, and the fragment collection changed shape. The skin to stone distance in the dolphin ranged from 7 to 8.5 cm on ultrasound and 5.5–7 cm in the harbor seal and was larger in CT images. BWL was attempted with human systems because these marine mammals have comparable skin to stone depths as humans. Long term, our urologists and veterinarians are working to address stone formation with diet [2]. Our experience here was failure of treatment with the allopurinol was indication to seek other therapies.

BWL provided a minimally invasive option for treating renal and ureteral stones in marine mammals. Development and trials are beginning for pet dogs and in particular pet cats because they similarly have few options for obstructing ureteral stones [20]. Animal treatments also provide ways to learn about and possibly improve human treatment; for instance, follow-up with humans is usually a week to 3 months later, whereas the mammals were watched round the clock with at least weekly ultrasound imaging. Lastly, these results add to growing data on the potential of BWL and ultrasonic propulsion to move stone intervention forward in the care cycle by treating ureteral stones on first presentation and prophylactically treating even small renal stones [17, 21].

## Supplementary Information

Below is the link to the electronic supplementary material.

Supplementary file1 (DOCX 13 KB)

Supplementary file2 (AVI 6836 KB)

Supplementary file3 (AVI 186784 KB)

Supplementary file4 (MP4 15,573 KB)

## Data Availability

All data supporting the findings of this study are available within the paper and its Supplementary Information.
