# Supplementary material for: Application of novel burst wave lithotripsy and ultrasonic propulsion technology for the treatment of ureteral calculi in a bottlenose dolphin (Tursiops truncatus) and renal calculi in a harbor seal (Phoca vitulina)
Source: Urolithiasis. 2024 Jan 8;52(1):21. doi: 10.1007/s00240-023-01515-6 (PMC10774161; doi:10.1007/s00240-023-01515-6)
Supplement: Supplementary file 1 — Supplementary file1 (DOCX 13 KB) [file 240_2023_1515_MOESM1_ESM.docx]

**Legend of Supplemental Videos**

1. Supplemental Video 1: Display on the UW investigational device Propulse 1 during fragmentation of a 10-mm stone in water. The stone is seen to fragment and fragments to move. The display on the SonoMotion system is more refined.
2. Supplemental Video 2: Video of 8-mm stones fragmenting completely in 3 minutes. Small passage flakes steadily break off the stone from start to finish.
3. Supplemental Video 3: A 10-mm stone in the seal kidney seen to fragment on the SonoMotion Break Wave therapy system monitor during treatment.
